# Supplementary figures and images for: The Eucalyptus grandis chloroplast proteome: Seasonal variations in leaf development
Source: PLoS One. 2022 Sep 1;17(9):e0265134. doi: 10.1371/journal.pone.0265134 (PMC9436043; doi:10.1371/journal.pone.0265134)

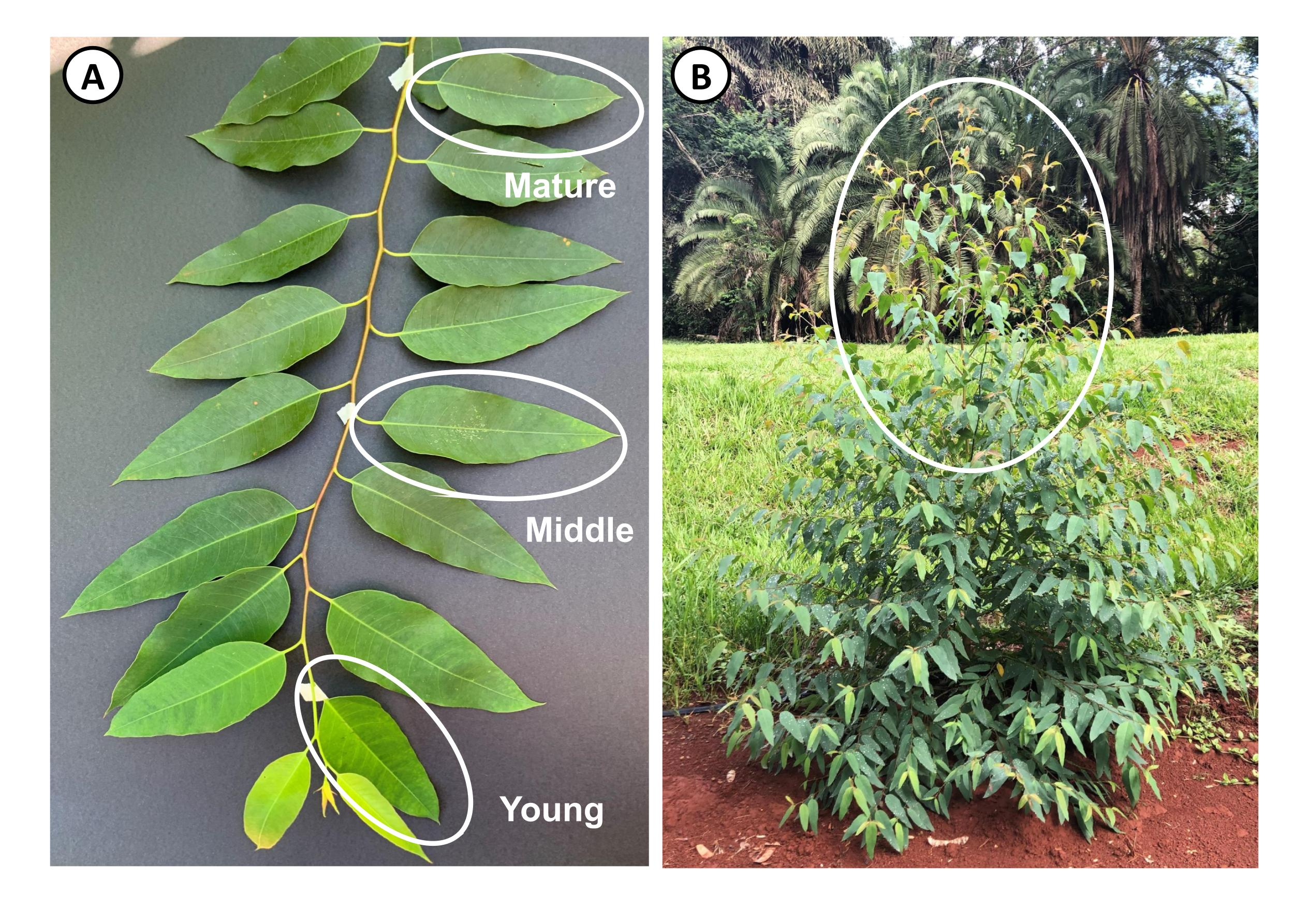

Supplement: S1 Fig — Leaf developmental assay, branches were divided into 3 different regions (young, middle, and mature), according to the fluorescence data (FV/FM) and chlorophyll relative quantification (CCI) (A). Proteome seasonal variation assay, forty leaves were collected from the first until the fifth node in all four seasons (Spring, Summer, Fall and Winter) (B). (TIFF) [file pone.0265134.s001.tiff]

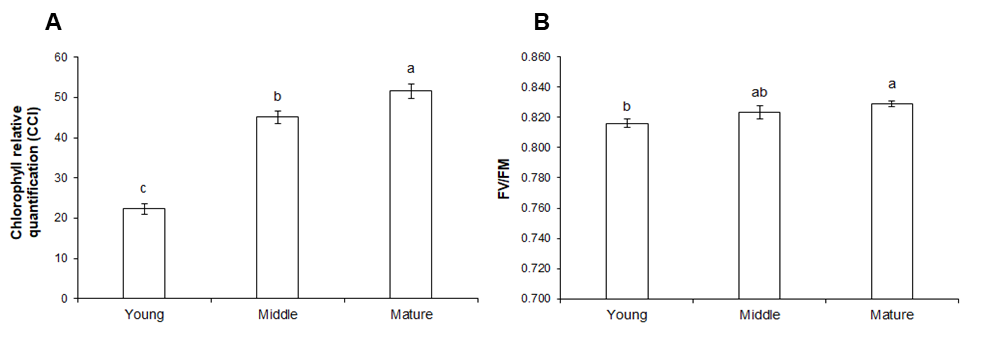

Supplement: S2 Fig — Chlorophyll relative quantification (CCI) (A) and Quantum efficiency of photosystem II (FV/FM) (B). Leaves were isolated from young, middle, and mature regions as described in the Material and Methods section. Different letters indicate significant differences according to Tukey’s test (p <0.05). (TIF) [file pone.0265134.s002.tif]
