# Supplementary material for: The Eucalyptus grandis chloroplast proteome: Seasonal variations in leaf development
Source: PLoS One. 2022 Sep 1;17(9):e0265134. doi: 10.1371/journal.pone.0265134 (PMC9436043; doi:10.1371/journal.pone.0265134)
Supplement: S3 Table — Proteins were classified according to their abundance profile: “upregulated”—proteins with the highest abundance in mature leaves; “downregulated”–proteins with the lowest abundance in young leaves; "undefined"–proteins with undefined profile pattern. (DOCX) [file pone.0265134.s005.docx]

S3 Table. Expression profile of the differentially regulated proteins identified in the Eucalyptus grandis chloroplasts during leaf development. Proteins were classified according to their abundance profile: “up-regulated” - proteins with the highest abundance in mature leaves; “down-regulated” - proteins with the lowest abundance in young leaves; "undefined" - proteins with undefined profile pattern.

| ***Eucalyptus grandis* accession ^a^** | **Sequence coverage (%)^b^** | **MS/MS count ^c^** | **LFQ intensity (log2) ^d^** | | | **p-value^e^** | **Description ^f^** | **Protein expression ^g^** |
| --- | --- | --- | --- | --- | --- | --- | --- | --- |
|  |  |  | *Young* | *Middle* | *Mature* |  |  |  |
|  |  |  |  |  |  |  |  |  |
| **up-regulated** |  |  |  |  |  |  |  |  |
| Eucgr.A01135.1 | 12.90 | 27 | 23.46 | 23.97 | 23.98 | 0.0030 | Isocitrate dehydrogenase V (IDH-V) | 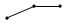 |
| Eucgr.K02742.4 | 9.30 | 13 | 21.12 | 21.78 | 21.96 | 0.0397 | Mitochondrial pyruvate carrier (MPC1) | 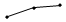 |
| Eucgr.D00854.1 | 46.30 | 832 | 30.62 | 30.82 | 31.22 | 0.0154 | Photosystem II subunit Q-2 (PSBQ-2) | 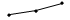 |
| Eucgr.F00373.3 | 23.30 | 105 | 25.37 | 25.74 | 26.40 | 0.0022 | Ascorbate peroxidase 4 (APX4) | 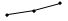 |
| Eucgr.F01793.1 | 42.70 | 498 | 29.46 | 29.44 | 29.87 | 0.0267 | Glyceraldehyde-3-phosphate dehydrogenase B subunit (GAPB) | 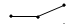 |
| Eucgr.F03445.1 | 13.30 | 4 | 20.87 | 20.51 | 22.08 | 0.0109 | Chloroplastic lipocalin (CHL) | 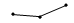 |
| Eucgr.F03557.1 | 26.90 | 163 | 26.47 | 26.96 | 27.35 | 0.0368 | Catalase 2 (CAT2) | 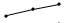 |
| Eucgr.F04344.2 | 12.30 | 30 | 23.36 | 23.80 | 24.44 | 0.0004 | Thylakoidal ascorbate peroxidase (tAPX) | 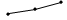 |
| Eucgr.G02623.1 | 11.70 | 9 | 22.06 | 23.04 | 23.18 | 0.0131 | Ferredoxin/thioredoxin reductase subunit A2 (FTRA2) | 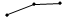 |
| Eucgr.H00144.2 | 36.30 | 192 | 26.92 | 27.79 | 28.17 | 0.0015 | Plastid-lipid associated protein PAP/fibrillin family protein (FIB4) | 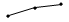 |
| Eucgr.H03311.1 | 41.10 | 151 | 26.97 | 27.09 | 27.25 | 0.0297 | Chloroplast RNA binding (CRB) | 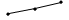 |
| Eucgr.H04456.1 | 32.00 | 239 | 27.29 | 27.70 | 28.00 | 0.0364 | Thylakoid lumen 18.3 kDa protein (TLP) | 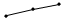 |
| Eucgr.I01025.1 | 57.40 | 1384 | 31.14 | 31.52 | 32.01 | 0.0260 | Photosystem II subunit O-2 (PSBO-2) | 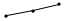 |
| Eucgr.I01790.1 | 47.20 | 682 | 28.92 | 29.98 | 29.94 | 0.0003 | Carbonic anhydrase 1 (CA1) | 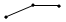 |
| Eucgr.J00682.1 | 9.90 | 22 | 21.10 | 23.03 | 23.27 | 0.0385 | Pyrimidine 2 (PYD2) | 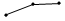 |
| Eucgr.K00110.7 | 10.50 | 36 | 23.70 | 24.44 | 24.97 | 0.0009 | Fe superoxide dismutase 2 (SODFe2) | 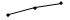 |
| Eucgr.L02773.1 | 43.40 | 381 | 27.75 | 28.30 | 28.46 | 0.0105 | Glycine cleavage T-protein family (GCT) | 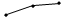 |
|  |  |  |  |  |  |  |  |  |
| **down-regulated** |  |  |  |  |  |  |  |  |
| Eucgr.A00348.1 | 6.90 | 9 | 23.45 | 20.06 | 19.95 | 0.0002 | Lipoamide dehydrogenase 1 (LPD1) | 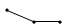 |
| Eucgr.A00746.1 | 21.80 | 10 | 23.02 | 21.64 | 20.35 | 0.0026 | Lipid transfer protein 1 (LTP1) | 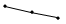 |
| Eucgr.A01783.2 | 15.60 | 6 | 22.55 | 20.54 | 20.62 | 0.0032 | Isopentenyl pyrophosphate pyrophosphate isomerase (IPP2) 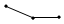 | |
| Eucgr.A02177.1 | 5.90 | 4 | 21.96 | 20.37 | 19.46 | 0.0366 | Dicarboxylate diiron protein, putative (CRD1) | 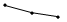 |
| Eucgr.A02448.1 | 5.40 | 5 | 22.06 | 20.63 | 20.74 | 0.0228 | NAD(P)-binding Rossmann-fold superfamily protein (ENR1) | 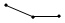 |
| Eucgr.B02310.1 | 49.40 | 87 | 26.13 | 25.45 | 24.64 | 0.0017 | Rubisco activase (RCA) | 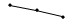 |
| Eucgr.B02864.1 | 10.60 | 7 | 23.33 | 20.52 | 20.27 | 0.0008 | Aldolase superfamily protein (ALD) | 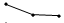 |
| Eucgr.B03013.1 | 39.10 | 41 | 25.75 | 24.18 | 23.23 | 0.0009 | Ribulose bisphosphate carboxylase (small chain) (RBCS1) | 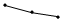 |
| Eucgr.C03812.2 | 29.00 | 52 | 24.25 | 22.85 | 22.69 | 0.0155 | Thioredoxin-dependent peroxidase 1 (TPX1) | 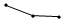 |
| Eucgr.F01098.1 | 14.10 | 26 | 24.93 | 23.64 | 22.60 | 0.0473 | Protochlorophyllide oxidoreductase A (PORA) | 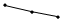 |
| Eucgr.F01365.2 | 8.60 | 40 | 24.14 | 23.80 | 23.57 | 0.0082 | Ribosomal protein L13 family protein (RPL13) | 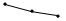 |
| Eucgr.F02457.3 | 15.20 | 9 | 23.78 | 20.44 | 20.29 | 0.0000 | Tetratricopeptide repeat (TPR)-like superfamily protein (TPR) | 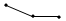 |
| Eucgr.F02835.1 | 5.50 | 9 | 22.43 | 21.97 | 20.45 | 0.0118 | pfkB-like carbohydrate kinase family protein (FKB) | 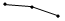 |
| Eucgr.F03433.1 | 8.60 | 21 | 23.19 | 22.46 | 19.84 | 0.0067 | Thiazole biosynthetic enzyme, chloroplast (THI1) | 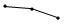 |
| Eucgr.F03789.1 | 29.70 | 394 | 29.22 | 28.66 | 28.76 | 0.0002 | Chlorophyll A-B binding family protein (PSBS) | 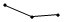 |
| Eucgr.G01124.1 | 17.90 | 24 | 24.14 | 20.68 | 20.82 | 0.0019 | Pyridoxal phosphate dependent transferases superfamily (PLP) | 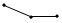 |
| Eucgr.G03333.2 | 13.70 | 61 | 25.26 | 24.61 | 24.34 | 0.0130 | Ribosomal protein L1p/L10e family (PRPL1) | 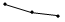 |
| Eucgr.H00128.2 | 4.30 | 3 | 22.03 | 19.71 | 20.22 | 0.0099 | RNA-binding (RRM/RBD/RNP motifs) family protein (RBD1) | 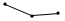 |
| Eucgr.H02992.2 | 6.50 | 18 | 25.07 | 20.21 | 20.02 | 0.0003 | Plastid developmental protein DAG (DAG) | 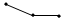 |
| Eucgr.H04084.2 | 14.40 | 56 | 25.39 | 24.64 | 24.68 | 0.0295 | Ribosomal protein S1 (RPS1) | 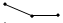 |
| Eucgr.I01803.1 | 12.70 | 15 | 25.69 | 20.56 | 21.57 | 0.0006 | Oxidoreductase, zinc-binding dehydrogenase family protein (AOR1) | 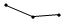 |
| Eucgr.I01807.1 | 38.40 | 54 | 26.24 | 21.16 | 21.04 | 0.0001 | Oxidoreductase, zinc-binding dehydrogenase family protein (AOR2) | 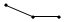 |
| Eucgr.I02771.1 | 28.80 | 529 | 28.93 | 28.58 | 28.46 | 0.0415 | Chloroplast heat shock protein 70-2 (HSC70-2) | 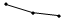 |
| Eucgr.J01250.1 | 7.60 | 29 | 24.68 | 24.10 | 24.15 | 0.0110 | RNA-binding (RRM/RBD/RNP motifs) family protein (RBD2) | 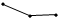 |
| Eucgr.J02957.3 | 27.40 | 6 | 22.68 | 20.49 | 20.54 | 0.0042 | Curvature thylakoid 1C (CURT1C) | 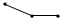 |
| Eucgr.K00727.1 | 14.80 | 44 | 25.24 | 24.33 | 22.59 | 0.0195 | Glutamate-1-semialdehyde-2,1-aminomutase (GSA1) | 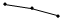 |
| Eucgr.K01283.1 | 26.10 | 14 | 24.79 | 20.65 | 20.57 | 0.0002 | Lipid transfer protein 1 (LTP2) | 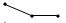 |
| Eucgr.K02198.1 | 25.20 | 176 | 27.02 | 26.58 | 26.51 | 0.0023 | CLPC homologue 1 (CLPC1) | 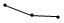 |
| Eucgr.K02223.1 | 47.50 | 473 | 30.75 | 30.36 | 30.20 | 0.0357 | Ribulose bisphosphate carboxylase (small chain) (RBCS2) | 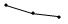 |
|  |  |  |  |  |  |  |  |  |
| **Undefined** | |  |  |  |  |  |  |  |
| Eucgr.A01933.1 | 30.80 | 242 | 27.37 | 26.88 | 27.07 | 0.0188 | FtsH extracellular protease family (FTSH) | 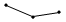 |
| Eucgr.A02930.1 | 18.90 | 25 | 23.29 | 23.96 | 23.58 | 0.0059 | Nucleoside diphosphate kinase family protein (NDPK1) | 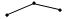 |
| Eucgr.B00512.1 | 7.60 | 15 | 23.71 | 19.78 | 21.90 | 0.0060 | Pyridine nucleotide-disulphide oxidoreductase family protein (CHLP) | 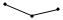 |
| Eucgr.D00321.1 | 40.30 | 145 | 26.57 | 25.95 | 26.08 | 0.0274 | Light-harvesting chlorophyll-protein complex II subunit B1 (LHB1) | 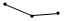 |
| Eucgr.E00068.1 | 23.90 | 120 | 26.27 | 25.77 | 25.88 | 0.0214 | Thylakoid rhodanese-like (TROL) | 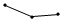 |
| Eucgr.E02392.1 | 10.40 | 42 | 24.71 | 24.39 | 25.03 | 0.0268 | NAD(P)-binding Rossmann-fold superfamily protein (ENR2) | 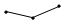 |
| Eucgr.F01905.1 | 6.10 | 6 | 22.16 | 20.16 | 21.40 | 0.0027 | Outer envelope pore protein 24, chloroplastic-like (OEP24A) | 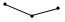 |
| Eucgr.G01465.1 | 2.40 | 4 | 21.71 | 20.19 | 20.91 | 0.0350 | Presequence protease 2 (PREP2) | 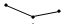 |
| Eucgr.G01561.1 | 11.80 | 50 | 23.51 | 24.32 | 24.21 | 0.0256 | Eukaryotic aspartyl protease family protein (ASP) | 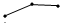 |
| Eucgr.H04427.1 | 12.10 | 18 | 23.42 | 20.82 | 22.39 | 0.0451 | Dehydroquinate dehydratase/ shikimate dehydrogenase (MEE32) | 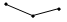 |
| Eucgr.I02231.1 | 5.40 | 5 | 22.05 | 19.62 | 20.41 | 0.0430 | Phosphoserine aminotransferase (PSAT) | 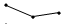 |
| Eucgr.J00025.2 | 23.00 | 28 | 24.07 | 23.52 | 23.99 | 0.0015 | Heat shock protein 70 (HSP70) | 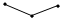 |
| Eucgr.K00389.1 | 32.60 | 380 | 29.28 | 29.27 | 29.73 | 0.0341 | Photosystem I subunit F (PSAF) | 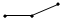 |
| Eucgr.K02786.1 | 21.50 | 200 | 27.33 | 26.81 | 27.05 | 0.0206 | RAB GTPase homolog E1B (RABE1B) | 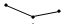 |
| Eucgr.L02980.1 | 37.30 | 68 | 23.04 | 26.84 | 21.82 | 0.0455 | Heat shock protein 21 (HSP21) | 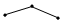 |

^a^ Protein identifier according to the *E. grandis* v 2.0 annotation

^b^ Percentage of coverage of the proteoform identification

^c^ Number of MS / MS spectra identified

^d^ Relative protein expression in each leaf tissue represented by the mean of LFQ intensity transformed into log (2)

^e^ p-value of statistical analysis

^f^ Functional Annotation

^g^ Protein expression trend
