# Supplementary material for: The Eucalyptus grandis chloroplast proteome: Seasonal variations in leaf development
Source: PLoS One. 2022 Sep 1;17(9):e0265134. doi: 10.1371/journal.pone.0265134 (PMC9436043; doi:10.1371/journal.pone.0265134)
Supplement: Attachment — Submitted filename: Baldassi_2022_Response_Reviewers_R1-ab-tsb-ab.docx [file pone.0265134.s007.docx]

**The *Eucalyptus grandis* chloroplast proteome: leaf development and seasonal variations**

Amanda Cristina Baldassi and Tiago Santana Balbuena

**Response to Reviewers**

**Reviewer #1**

Major concerns

[1] A major drawback of this study is the purity of the chloroplast fraction. On what basis the authors made the statement “…...that our chloroplast isolation and enrichment strategy was effective in reducing the number of cross-contaminants…...” (Results and Discussion: Line 259-262; Final Remark: Line 679-682). Use of NSAF approach for determining organelle abundance typically rely on detection of a very few marker proteins, and hence is unsatisfactory. In my opinion, there should be a few more more wet-lab experiments that would justify the findings of proteomics analysis. The authors should pick up a few candidates and examine their translocation the chloroplasts, say by transient expression analysis.

Response:We understand the concerns regarding this issue. In addition to the data described in the manuscript, the supplementary material 2 of one of our group’s publication (https://doi.org/10.1007/s00468-018-1750-8) gives support to our chloroplast isolation procedure. We did not carry out a Western Blot Immuno assays to confirm results as the fragment ions from MS/MS data and stringency used for protein identification give support for the identifications proposed in the manuscript. We also used 2 different quantification metrics (NSAF and LFQ intensity) to confirm the decrease in abundance of the contaminants. However, we agree that the sentence “…...that our chloroplast isolation and enrichment strategy was effective in reducing the number of cross-contaminants…...” have to be rephrased since Table 1 showed reduction in the abundance of the contaminants but not in the number contaminant IDs.

[2] The manuscript describes some interesting aspects of biochemistry operating in chloroplast proteome. The authors’ preparation of chloroplast yields a considerable number of unexpected proteins, which are classically considered to be translocated to other cellular compartments. Since contamination from other organelles cannot be discounted, the information obtained for the unexpected proteins in the fraction remains highly suspected. The authors should consider revising the manuscript by focusing on chloroplast proteins, and reducing the description and discussion of the unexpected proteins to a minimum.

Response:Reviewer is correct, even though we used a chloroplast isolation procedure, we indeed identified a considerable number of unexpected proteins. However, it should be highlighted that we only discussed data from proteins whose cellular location was predicted to be chloroplatic. Identified proteins that were not predicted to be localized in the chloroplast were not taken into consideration in our analysis and discussion. .

[3] The authors stated, in the abstract, that “This study contributes to a more comprehensive understanding on the subcellular mechanisms that lead to plant leaf adaptation and ultimately to Eucalyptus grandis productivity”. There is a need to revisit the statement because it is not convincing in the case of Eucalyptus grandis productivity.

Response: Reviewer is correct. The statement was rephrased.

[4] Eucalyptus leaves are a great source of antioxidants, particularly flavonoids, which are effective in protection against oxidative stress and ROS damage. I didn’t find any mention of this sort in the manuscript. Instead, the first two paragraphs of the Introduction contain too much general information which are text book materials. I feel that these paragraphs can be clubbed and restructured. Noticeably, I have counted as many as 41 references in the Introduction, many of which describe the general aspects of chloroplast biogenesis and the function of chloroplast in plant. The number of references in this section should be reduced.

Response:Reviewer is correct. The paragraphs were restructured.

[5] “…… 50 μg of proteins were separated for 1 hour by denaturing electrophoresis on a polyacrylamide gel containing sodium dodecyl sulfate (SDS-PAGE) to obtain a single band”. What is the meaning of “to obtain a single band”?

Response: The goal of loading the chloroplast extracts on SDS-Page gel was to clean up samples from salts, denaturants, detergents and other buffer components that could interfere in the MS analysis. We only allowed 1 cm of samples to enter into the gel and excised the entire segment as a single band as you can see in the picture below:

[6] In general, experimental protein localization requires a great deal of time and resources, which are bypassed by prediction analysis. It is recommended that the prediction analysis be accomplished by a series of prediction tools for cross-validation test. In this study, subcellular localization of the proteins was predicted by ChloroP and Predotar. Why don’t add some other tools, for example, BaCelLo, WoLF PSORT, TargetP etc.

Response: We used two different prediction tools: ChloroP that uses chloroplast transit peptide and Predotar that uses N-terminal targeting sequences to predict chloroplast proteins. Instead of adding other prediction tools into the pipeline, we combined data from those tools with data from chloroplast protein sequence databases (PPDB and AT-Chloro) in order to identify the chloroplast proteins described in the manuscript. Although not used in our research, we evaluated the potential gains of adding other prediction tools. As we can see below, contribution of other prediction tools is limited to approximately 10% of the current identifications and, thus, were not incorporated into our work.

Minor concerns

Authors should ensure the text is polished enough to be published. The English needs to be much improved and the authors should request the assistance of a professional service. While the entire text requires revision, I will cite some issues that should be addressed to avoid errors and misinterpretations. Please note that the revision should not be limited to the following items:

[1] Please carefully edited the manuscript; remove most acronyms unless very much necessary.

Response: We removed them.

[2] “…...may contribute to enlarge the understanding…...” (Abstract) is not a good read. Similarly, “…… proteome analysis of the Eucalyptus grandis chloroplast proteome….”. These should be suitably rephrased.

Response: We did it.

[3] There must be a space between the numerical value and unit symbol; missing at so many places in the text.

Response: We corrected it.

[4] “Eucalyptus grandis were planted” is more appropriate than “Eucalyptus grandis plants were cultivated” (Materials and Methods: Line 108).

Response: We corrected it.

[5] The sentence “For both assays using leaf sampling, plant material………chloroplasts, as described in the next sections” (Line 124-125) is absolutely unnecessary.

Response: We removed the sentence.

[6] Rephrase the sentence “Fluorescence analysis was performed on the fluorometer FluorPen FP …………” (Line 130-132).

Response: We rephrased accordingly.

[7] “Aliquots of six grams of leaves...” is imperfect in the sentence (Line 143-144).

Response: We corrected it.

[8] “……seems prone to catabolic and redox processes as leaves age….” (Line 683-684) doesn’t make sense.

Response: We corrected it.

[9] Fig. S2 legend: Which one is correct: chlorophyll relative quantification (CCI) or chlorophyll content index (CCI)?

Response:Chlorophyll relative quantification is correct. We corrected in the manuscript.

[10] “up-regulated” and “down-regulated” can be replaced with “upregulated” and “downregulated”, respectively throughout the text.

Response: We replaced the term throughout the text.

[11] References need extensive corrections since the references are not in the same format [as per the style of PLoS ONE], particularly the journal names (Reference 33: Environ. Sci. Pollut. Res.; Reference 33: J Exp Bot.).

Response: References were revised and corrected.

**Reviewer #2**

I have some concern on the possibility to excise a single band from an SDS-PAGE gel where 50 ug of proteins have been loaded. Often, using a 2D-PAGE overlapping of bands occurs.

Response: As we answered to the reviewer #1, the goal of loading the chloroplast extracts on SDS-PAGE gel was to clean up samples from salts, denaturants, detergents and other buffer components that could interfere in the MS analysis.

I invite the authors to be consistent with Figs and Tables’ nomenclature (e.g., 2S vs S2)

Response: We did it.

Figure S2B is a duplication of S1A
Response: The figure S2 was modified. We decided to keep only S2A with CCI and FV/FM results.

In all the text many words are collapsed with spaces missing (e.g., L59, 64, 70…304, 351, 518, 527, 550 etc)

Response: True. We revised this issue throughout the text.

L341-63 and 440-53, please, insert the full protein name (as authors do in L413-419)

Response: The full protein names were added in the Supplementary material (S3 Table).

L352 and 451 “PRPL1” is a typo?

Response: True. Thanks for pointing this out. We revised this issue throughout the text.

L424-425 this sentence is not clear

Response: We tried to clarify the sentence.

L413-19 and 433-37 short name of proteins should be near their full name

Response: We corrected this issue.

Please, pay attention to typos (e.g., L580 “the” is duplicated, “fakk” should be “fall”)

Response: We revised the manuscript in order to find and correct typos.
