# Supplementary material for: The Eucalyptus grandis chloroplast proteome: Seasonal variations in leaf development
Source: PLoS One. 2022 Sep 1;17(9):e0265134. doi: 10.1371/journal.pone.0265134 (PMC9436043; doi:10.1371/journal.pone.0265134)
Supplement: Attachment — Submitted filename: Baldassi_2022_Response_Reviewers_R2_tsb.docx [file pone.0265134.s008.docx]

**RESPONSE TO REVIEWERS**

**The *Eucalyptus grandis* chloroplast proteome: seasonal variations in leaf development**

Amanda Cristina Baldassi, Tiago Santana Balbuena

**Reviewer #1:**

Compared to the original manuscript, the revised version has been greatly improved, especially with respect to structural rearrangement and copyediting. The authors addressed most of the reviewers' questions and comments. Yet I have few concerns which are listed below:

[1]. I would prefer a modified title “The Eucalyptus grandis chloroplast proteome: seasonal variations in leaf development” and over “The Eucalyptus grandis chloroplast proteome: leaf development and seasonal variations over”.

>>Authors: We changed the MS title according to this suggestion.

[2]. The last line in the abstract “Mass spectrometric data are available via ProteomeXchange under identifier PXD029004” can be moved to “Results and Discussion” section.

>>Authors: We moved the sentence to the “Results and Discussion” section.

[3]. “Transient seasons” in the subtitle “Transient seasons induce the most pronounced chloroplast proteome changes” is not a good read and should be modified.

>>Authors: We incorporated this change.

[4]. The phrase “Proteome isolation” is not appropriate and hence the subtitles “Chloroplast proteome isolation” and “Total leaf proteome isolation” should be modified.

>>Authors: True. Thanks for pointing this out. We changed the phrase.

[5]. Subtitle “Final Remarks” can be replaced with “Conclusions”.

>>Authors: We incorporated this change.

We thank both Reviewers for their suggestions and critics. Thank you for using your time and energy to make it a better manuscript.
